# Moon and Health: Myth or Reality?

**DOI:** 10.7759/cureus.48491

**Published:** 2023-11-08

**Authors:** Mihika V Gokhale, Sunil Kumar

**Affiliations:** 1 Department of Medicine, Jawaharlal Nehru Medical College, Datta Meghe Institute of Higher Education and Research, Wardha, IND

**Keywords:** moon and psychiatry, full moon and health, epilepsy, lunar hypothesis, lunar phases

## Abstract

Over the years, superstitions and myths have persisted regarding moon’s impact on human health and behaviour. Because of these myths and superstitions, several diseases that can be cured with effective management have been subjected to violent reactions from various communities. It becomes extremely important to unearth the facts related to these myths. It is important to study and deduce whether there is any notable relation that exists between moon and health of a human being. Neurological and psychological aspects have been dealt with more sensitivity since these are often related with taboos that exist in various communities. This study is mainly to review various research studies conducted in various health aspects including the cardiovascular system, neurology, psychiatry, birth rates, menstruation, sleep, injuries, crisis calls, and complications during surgeries.

## Introduction and background

The moon, the closest celestial body to Earth, has several myths around it. These myths have been there for years and have existed across the globe [1]. Superstitions regarding the moon are still prevalent worldwide. For understanding the concept of lunar hypothesis, we need to first understand what a lunar phase is. The figure of the illuminated part of the moon seen by an observer on Earth is the lunar phase [1]. A synodic month is the time interval between two lunar phases, which is 29.53 days on average [1]. There have been beliefs that the moon affects human behaviour in various ways. Neurological and psychological behaviours are particularly considered due to some demonic possession or punishment for a certain sin. Another belief that has persisted for several years is that of the metamorphosis of humans into wolves [2]. It has attracted attention, especially after the development of modern psychiatry and behavioural neurology [2]. It has been suggested by several writers that lycanthropes may be suffering from schizophrenia or there may be a history of ingestion of hallucinogens which can be voluntarily or involuntarily [2]. Ictal and interictal manifestation of complex partial seizures (also known as focal impaired awareness seizures or focal seizures) can explain the features of werewolf-like behaviour [2]. Those who either declared themselves as a werewolf or were suspected to be one were persecuted [2]. This metamorphosis was considered to be aggravated with different lunar phases [2]. There are studies which have shown that features of porphyria, hypertrichosis and pigmentation together can present a description of werewolves that is mentioned in older literature [3]. This disease, congenital porphyria can be characterised by severe photosensitivity where light exposure produces vesicular erythema, reddish brown urine, and tendency of the skin lesion to ulcerate [3]. Also in the areas that are photosensitive, pigmentation and hypertrichosis may develop [3]. Hence to avoid the exacerbation of their symptoms sunlight was often avoided, and they usually preferred night to wander around [3]. But all these features collectively framed a different picture and led to various stigmas and myths in the community. Most people across the globe have a notion that the moon has certain influence on human health and behaviour [1]. Despite the efforts of various researchers to provide scientific explanations against these myths or beliefs, people still believe that these myths are real. Hence, it becomes extremely important to prevent the spread of certain myths that are not supported by strong evidence but still prevail in different communities across the globe. Several research studies also suggest that the moon might affect human health but more conclusive evidence needs to be present so as to manage the condition scientifically. Disorders that have become taboo due to the already existing myths including epilepsy and psychiatric conditions need a scientific approach which would be beneficial for the management of this condition. This study mainly highlights scientific evidence to get a broader idea about the link that is present between the moon and human health if at all it exists. 

## Review

Method

**Figure 1:**
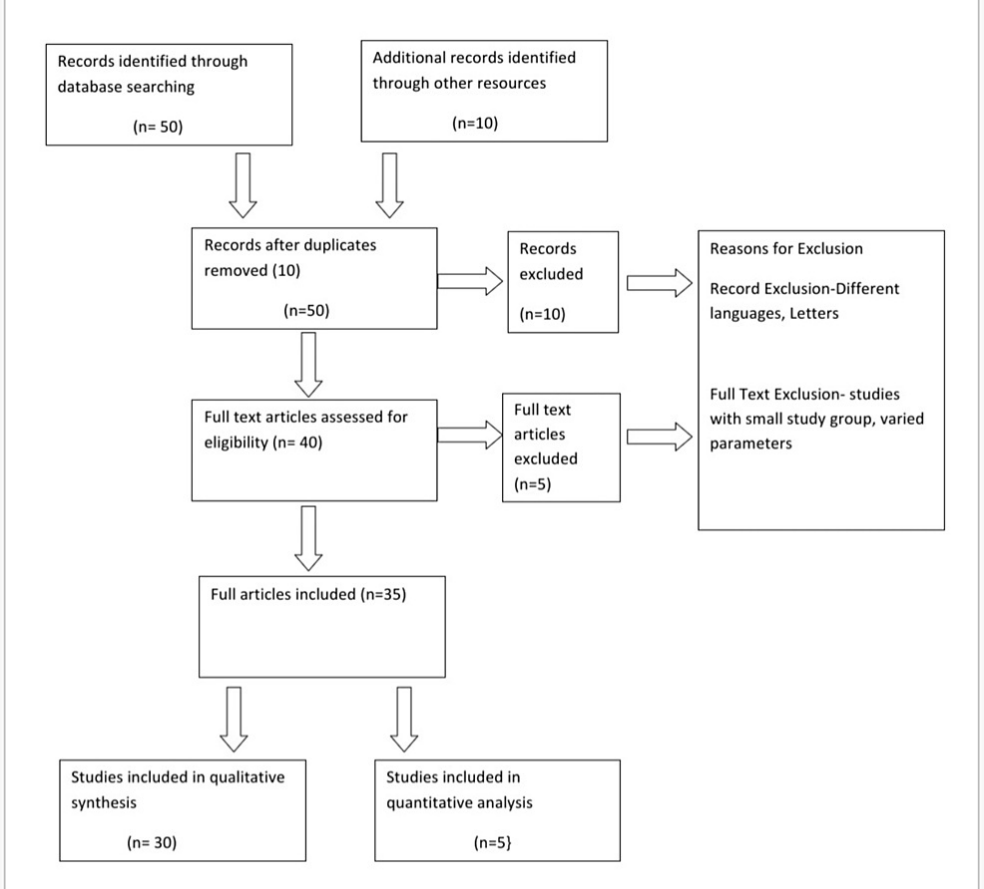
Flow Diagram for Study Selection Image Credits: Mihika V. Gokhale

For this study, objectives were set, one of which was to study the relationship between moon and body if at all it is present. Data was collected from the scientific literature which is present in various databases. For this, various articles were reviewed and analysed. Various databases like Google Scholar and PubMed were used for a detailed and focussed literature review. The terms that were used were “neurological effects of moon”, “psychiatric effects of moon”, “moon and human behaviour”, “moon and birth rates”, “menstruation and moon”, “sleep and moon”, etc. Articles were selected, and a detailed review was done along with the listing of these references. The entire process is illustrated in Figure 1.

Various aspects of human health

**Figure 2:**
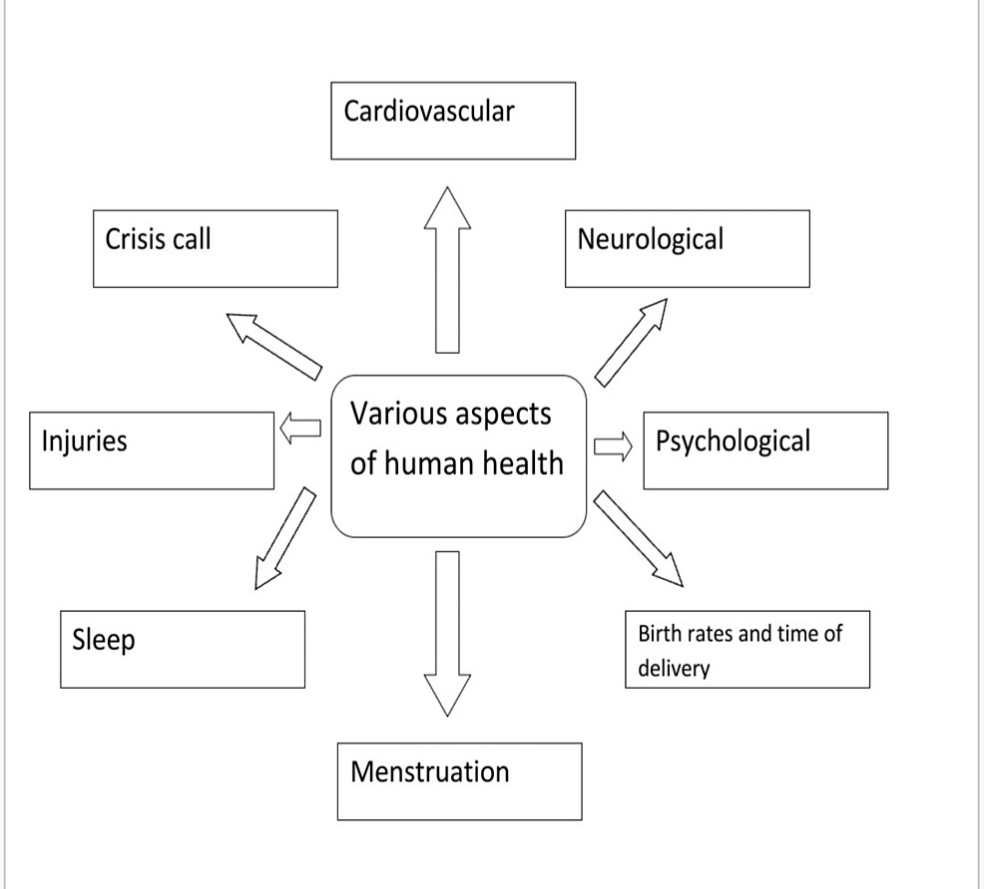
Few Health Aspects Discussed in the Review Image Credits: Mihika V. Gokhale

The moon has always attracted attention not only because of its astronomical importance but also because of the various mythologies associated with it. There has been a myth that has existed over the years that the moon has a role to play when it comes to human health. Several scientific explanations have been given to contradict these beliefs, but these are still prevalent. Various studies have shown that the full moon affects the various aspects of health in a human being [1]. These include the effects on the heart, on sleep, on birth rates and time of delivery, on menstruation, and on psychological aspects and neurological aspects of human health. Some human health aspects that are discussed in the review are illustrated in Figure 2. 

Cardiovascular Disorders

Heart diseases are very common in these years due to various reasons. Some of them are Myocardial Infarction, hypertension etc. Various etiological factors have been noted for the development of various cardiovascular diseases. These include age factors, lack of physical activities, obesity, smoking, alcohol and many more. However, certain studies have shown that there is a relationship between the moon’s gravitation and incidences of myocardial infarction and also regarding plaque rupture. Myocardial infarction is a problem across the globe. Various etiologies have been reported to cause myocardial infarction. Certain studies have also shown that rupture of plaque in the morning hours accounts for the circadian variation of acute myocardial infarction (AMI) [4]. There are studies that suggest, the gravitation of the moon may relate the AMI occurrences [5]. Researchers have also tried to study the consequences of seasons and the lunar cycle on the outcomes in hospitals after an ascending aortic dissection repair. They showed that there was no effect of seasons on the mortality following the repair. The full moon cycle gave the impression to decrease the odds of death and also with being a male, in need of a concomitant cardiac procedure was related to a shorter length of stay [6].

Neurological Disorders

Neurology has a complex role when it comes to the lunar cycle’s effect on it. The most dreaded affliction in the history of mankind is epilepsy. Even after various scientific efforts to explain the science behind epilepsy, it is still considered a demonic possession or a punishment offered by some supernatural powers [7]. There are several unfounded beliefs regarding the consequences of the moon on various medical conditions including epilepsy. Recent studies on the moon’s influence on seizures have given varied results. A study was conducted to study how the lunar cycle affects seizures suggested that it is the moon’s offering to the nocturnal luminance and the night brightness that influences the occurrence of seizures, not the lunar phase per se [8]. There were studies that were conducted to identify whether the sudden unexplained death in epilepsy has an association with the geomagnetic activity. In this study, it was found that there was nothing that supported the hypothesis that the occurrence of sudden unexplained death is associated with geomagnetic activities [9]. Certain studies were also conducted to assess a link between the frequency of the seizures and the full moon. For this, they reviewed the seizure occurrence that was noted in the epilepsy monitoring unit for a three-year period. It was deduced that there was no effect of the full moon on seizures as a whole, although it may affect non-epileptic seizures [10]. Over time, it has been debated whether lunar cycles affect seizures. There were questions about the potential reasons in those healthy individuals who present with their first-ever seizures. One of the reasons was the full moon. A study was conducted to study the impact of the lunar cycle on the first-ever unprovoked seizure occurrence. They analysed 1710 patients, and various subgroups and the cohort demonstrated that there was no notable variance in the occurrence of the seizures in the four lunar quadrants. It deduced that the lunar cycle does not influence the first unprovoked seizures [11]. Sudden unexpected death in epilepsy (SUDEP) is an important cause of the premature death of patients suffering from epilepsy, yet it is rare. A study was conducted to establish whether seasonality or lunar phase is a risk factor for sudden unexpected death in epilepsy. They found that there was no evidence that supported the idea of any link between the lunar phase and the number of sudden unexpected deaths in epilepsy [12]. Another study was conducted to assess the interaction between epilepsy and our circadian rhythm [13]. Here they found that circadian rhythm has an important role in seizure activities [13]. It was found that seizures occurred during the sleep state especially in the non-rapid eye movement (NREM) stage and deprivation of sleep after the morning affected or increased the risk of seizures [13]. Also intracranial aneurysm which is harboured by about 5% of the normal adult population is potentially lethal when it ruptures. There are beliefs that suggest that the new moon affects the incidence of intracranial aneurysm rupture. There was a study that was conducted to review whether there is a link between the rupture of the intracranial aneurysm and the lunar phase. It concluded that moon phases are not linked to the incidences of rupture of intracranial aneurysms [14].

Psychological Disorders

Health is not only about one’s physical health but it also includes one’s mental health as well. Several mental health aspects that are included in this study are suicidal tendencies, aggressive behaviours, depression, anxiety, mania and non-affective psychoses. There has been a myth that the moon affects the psychological aspects of a human being. The belief is still common [1]. This belief that human lives especially emotions are affected by the moon is widely accepted. It has existed for over a thousand years. These beliefs are largely immune to the fact that a lot of research and several studies have failed to support such beliefs. Lunatic, which is an antiquated and an offensive colloquial word, has a Latin origin (Luna - moon) [15]. The folklore that connects the full moon and the behavioural changes in a human is not easily explained by science but is prevalent and is observed. A study was conducted to observe the extreme behavioural disturbances that could be related to the full moon. It concluded that these behavioural disturbances are more common during the full moon [16]. Suicidal tendencies have increased over these years. Mostly these are related to stress or some other causes. However, the belief that there is an impact of lunar phases on suicides has been there for years. A population-based study was conducted to analyse the impact that the lunar phase has on suicides. It was observed through this study that there was no evidence that suggested a possible link between the lunar phase and suicides [17]. There were studies that were conducted to study the impact that lunar phases have on admission to psychiatric hospitals. The result of this study was that during the different moon phases, admission frequency did not differ significantly [18]. There was yet another study that was conducted to analyse the lunar effect on the consultation for anxiety and depression in general practice. It was found that there was no notable lunar effect when the patients with anxiety or depression consulted their general practitioners [19]. Another study was conducted in Goa. It mainly focussed on diagnoses like non-affective psychoses, depression and mania. This study was conducted to link these psychiatric disorders with full moon, new moon and other moon. It was noticed that there was a greater number of patients with non-affective psychoses on full moon days. For other disorders like mania and depression, this pattern was not observed [20]. There was also a study that was conducted to find out whether the psychiatric admissions or emergency evaluation had any link to the lunar cycle. It was found that there was no notable effect on the admission of patients with psychiatric illness by the lunar cycle [21].

Birth Rates and Time of Deliveries

It is a popular belief that the lunar cycle has a significant role in the deliveries. A study was conducted to identify a possible link between birth frequency and birth complications with lunar phases. It was found that there was nothing to suggest a possible association between them [22]. There was another retrospective observational study that was conducted to analyse the link between the lunar cycle and the frequency of deliveries. It was concluded that there was no evidence that suggested any relationship between birth frequency and the lunar cycle [23]. Another research was conducted to analyse the influence that the moon has on the timings of deliveries. In this study, it was observed that there was an influence, especially in multipara. But for the prediction for the days with the highest frequency of delivery, this is weak [24]. Another study was conducted to analyse the time distribution and spontaneous deliveries. As a result of this study, it was found that there existed a link between the lunar phases and the distribution of spontaneous deliveries [25]. Another study on the workload in the labour ward was conducted [26]. A total of 3706 spontaneous births occurred in the study period [26]. The analysis showed that on the full and at the new moon, there was no notable variance in the number of births [26].

Menstruation

Menstruation has been associated with a lot of taboos and myths across the globe. This has persisted for ages and it is still prevalent in many parts of the world. Several women suffer due to these existing myths. This is the reason why it becomes important to study the relationship between these myths and menstruation if at all it exists. As is known by all, a normal menstrual cycle is about 28 days and the lunar cycle is set to be about 29.5 days. There was a study conducted where 312 women were included and their menses records were maintained. Those women with menstrual cycle duration approaching the cycle of the moon tend to ovulate in the dark phase. Women who had irregular cycles also tended to ovulate in the dark phase [27]. Another study was conducted among the women of reproductive age group to study the relationship between the onset of the menstrual cycle, lunar phase and subjective sleep quality. There was no connection found between the onset of the menstrual cycle and lunar phases, but in the dark period menstrual cycle onset was linked with the worsening of the subsequent subjective sleep quality [28].

Sleep

Sleep is an extremely important phenomenon as it helps in the normal functioning of the brain. Abnormal sleeping patterns result in various disorders. In various cultures, it is thought that the moon has a significant influence on sleep. A study was conducted to study the influence on sleep duration because of the different phases of the moon [29]. 31 volunteers participated in the study and were examined and was deduced that subjective sleep varied with the lunar phases [29]. There was also evidence that suggested that there was an association between the rating of fatigue in the morning with the lunar cycle, where the full moon was related to more tiredness [29]. Another population-based study was conducted to study whether the moon affects sleep or not [30]. 2125 individuals were analysed in this population-based study [30]. It was concluded that there was no evidence that suggested a significant impact of the lunar cycle on human sleep [30]. Also, it was found that there is no notable variance in the cortisol levels in the various phases of the moon [30].

Injuries

Any harm caused to the body is an injury. Injuries can be fatal at times but can be managed if treated early. Often these injuries can be linked to various superstitions, but their relation needs intense study. Injuries can be caused due to various events including accidents, attacks etc. One of which can be a sports injury. It is extremely common in places where sports are encouraged. One of the most popular sports across the world is football. Football has a significant role to play because it not only keeps the player physically healthy but also keeps the player mentally healthy and disciplined. But everything comes with a price. Players are susceptible to injuries. These injuries include hamstring strain, ligament injury, ankle sprain etc. Most of the injuries occur mostly in the lower extremities, specifically in the ankles and the knees [31]. A study was conducted to find whether football injuries are linked to the lunar cycle or not [32]. It was deduced that gravitational pull, the full moon, and the new moon have no significant effect on football injuries [32].

Crisis Call and Lunar Cycle

Crisis call refers to the calls made to the police station, poison centres, etc at the time of stress or emergency. Most often stress results in making these crisis calls to various centres. There are beliefs that the lunar phase has a significant role to play during the time of stress. Various studies have been conducted to study this belief [33]. A time series analysis on 4575 crisis call was conducted to study the significance of the lunar hypothesis [34]. The evidence did not support the lunar hypothesis [34]. Another study reviewed 12 studies where they examined the link between the crisis calls to police stations, poison centres. etc. and the lunar cycle [35]. It was deduced that there was nothing substantial to support the belief that the lunar cycle has any relation with the frequency of crisis calls [35].

Intraoperative and Perioperative Complications

Blood loss is a common intraoperative complication that can occur due to various causes. It can be fatal at times and the patient might land up in shock. Various known causes have been scientifically proven. But is it related to the lunar cycle is a question that needs an answer. A study, where analysis was done to relate this myth with real-life surgeries was conducted [36]. The analysis suggested that moon phases, zodiac signs, or Friday 13th do not impact the intraoperative blood loss or emergency frequencies [36]. Another study was conducted to evaluate whether the lunar cycle had any impact on the peri-operative complications in a total hip arthroplasty [37]. There was no evidence that suggested this relationship [37]. More study needs to be done to review the analysis. The various studies that are included in the review are summarised in Table 1. 

**Table 1:** Summary of Included Studies in the Review

Authors	Year	Location	Findings
Roy et al. [1]	2017	Bangladesh	The review suggested that in most of the cases, there is no relationship found between the health and full moon.
Drake Jr [2]	1992	Columbus	Various aspects of lycanthropy that include medical and neuropsychiatry. Also it has been found that clinical description of illnesses that have a diabolic origin was a part of the evolution of Renaissance medicine to the scientifically based future from the Galenic past.
Illis L [3]	1964	UK	Features of porphyria together with hypertrichosis fit well in the description of werewolves in the older literature.
Tanaka et al. [4]	2004	Japan	The circadian variation observed in the Acute Myocardial Infarction is due to the increased incidences of rupture of the plaque in the morning.
Wake et al. [5]	2008	Japan	The moon's gravitation can have an effect on the Acute Myocardial Infarction occurrence.
Shuhaiber et al. [6]	2013	USA	Seasons showed following aortic dissection repair, no influence on mortality while the age of the patient significantly increases the odds of death.
Jilek‐Aall [7]	2005	Africa	Constant efforts in health education in the view of organized treatment for epilepsy can change in the popular notions related to epilepsy.
Baxendale et al. [8]	2008	UK	The brightness of the sky and the contribution that is made by the phases of the moon in nocturnal luminance may influence the epileptic seizures.
Schnabel et al. [9]	2000	Germany	Hypothesis was not supported by the study that the sudden unexplained death in epilepsy has an association with geomagnetic activity.
Benbadis et al. [10]	2004	USA	No effect of full moon on seizures at a whole was observed but there may be a possible effect on non-epileptic seizures.
Wang et al. [11]	2022	Australia	Lunar cycles do not influence the first unprovoked seizures.
Bell et al. [12]	2010	UK	No association was found in sudden unexpected death in epilepsy and month, season or lunar phase.
Liu et al. [13]	2022	China	Circadian rhythm has an important role in seizure activities.
Bunevicius et al. [14]	2017	Lithuania	Incidences of intracranial aneurysm rupture is not related to moon phases are not.
Gupta et al. [15]	2019	Switzerland	No evidence supports the belief that moon influences our mental health.
Calver et al. [16]	2009	NSW	Disturbances in the behaviour were observed more during the full moon.
Biermann et al. [17]	2005	Germany	No evidence suggested a possible relation between lunar phase and suicide.
Maslov [18]	2022	Russia	The admission frequency did not differ significantly in the various phases of moon.
Wilkinson et al. [19]	1997	UK	The moon had little effect, when patients consulted their general practitioner with depression or anxiety.
Parmeshwaran et al. [20]	1999	India	A link between the full moon and non-affective psychoses has been demonstrated.
McLay et al. [21]	2006	USA	Lunar phase was not associated in any significant way to the admissions in psychiatry.
Arliss et al. [22]	2005	USA	No influence of lunar cycle was observed on deliveries and complication.
Bharati et al. [23]	2012	India	The observation of the study did not support the hypothesis of an association of lunar cycle and frequency of deliveries.
Ghiandoni et al. [24]	1998	Italy	The study showed the impact of lunar phase on the time of delivery.
Ghiandoni et al. [25]	1998	Italy	A relation between lunar phases and distribution of spontaneous deliveries was found.
Joshi et al. [26]	1998	USA	There was nothing that suggested that when the full moon approaches, the number of births increases.
Cutler [27]	1980	California	Women with irregular cycles tend to ovulate in the dark phase.
Komada et al. [28]	2021	Japan	There was no connection found between the onset of the menstrual cycle and lunar phases, but in the dark period menstrual cycle onset was linked with the worsening of the subsequent subjective sleep quality.
Röösli et al. [29]	2006	Switzerland	There was an association of the rating of fatigue in the morning with the lunar cycle, where full moon was related with more tiredness.
Haba-Rubio et al. [30]	2015	Switzerland	There was no evidence that suggested a significant effect of lunar cycle on human sleep.
Dvorak et al. [31]	2000	Switzerland	Most of the injuries occur mostly in the lower extremities, specifically in the ankles and in the knees.
Yousfi et al. [32]	2018	Tunisia	Gravitational pull, full moon and the new moon have no significant effect on football injuries.
Kollerstrom et al. [33]	2003	UK	Beliefs that lunar phase has a significant role to play during the time of stress.
Wilson et al. [34]	1990	USA	The evidence did not support the lunar hypothesis regarding the crisis call.
Byrnes et al. [35]	1992	Canada	There was nothing substantial to support the belief that lunar cycle has any relation with the frequency of crisis calls.
Schuld et al. [36]	2011	Germany	Moon phases, zodiac signs, or Friday 13th does not impact the intra-operative blood loss or emergency frequencies.
Ficklscherer et al. [37]	2012	Germany	A study was conducted to study the lunar cycle had any impact on the peri-operative complications in a total hip arthroplasty, but no evidence suggested the hypothesis.

## Conclusions

In today’s world where medicine is supported by strong evidence, the belief that supernatural entities may be responsible for various aspects of human health still persists. Lunar phases have been subjected to various reviews for the same reason. In this review, we found that the possibility of the moon affecting human health although very less still exists. This possibility cannot be completely denied. Here it becomes extremely important to conduct research with adequate sample size so as to reach a significant conclusion. Hence, it is important to study and unearth the facts to make sure that these myths are supported by strong evidence if any. This will not only help in the scientific understanding of a particular condition but also in evidence-based treatment of it. Health education should also be encouraged to dispel the existing shame, guilt and anxiety that are related to several beliefs. Healthcare providers should be especially educated regarding these myths and superstitions and should have scientific knowledge of either in support or refusal of such myths because it will ensure evidence-based treatment and as a result, the outlook of patients for psychiatric and neurological conditions would change. 
